# The Association Between Stressful Social Roles and Sleep Quality Among Peri- and Post-Menopausal Women

**DOI:** 10.1093/geroni/igaf122.1362

**Published:** 2025-12-31

**Authors:** Luciana Giorgio, Erick Scherf, Michelle Favero

**Affiliations:** The University of Alabama, Tuscaloosa, Alabama, United States; The University of Alabama, Tuscaloosa, Alabama, United States; The University of Alabama, Tuscaloosa, Alabama, United States

## Abstract

Poor sleep, particularly during menopause, is associated with an increased risk of cognitive and cardiovascular disease in women’s later life. Although hormonal changes partially explain poor sleep quality during menopause, stress from performing multiple social roles may exacerbate poor sleep quality. This study examines the association between the number of social roles perceived as stressful and sleep quality among midlife women. Using survey data from peri/post-menopausal women participating in the Study of Women Across the Nation Wave 1 (n = 1,690), we conducted linear regressions to test the effects of social role stress on sleep quality. The number of social roles identified as stressful were summed across social role categories (e.g., employment, family caregiving, being a spouse/partner, and childrearing). Sleep quality was measured using the Women’s Health Initiative Insomnia Scale (WHIIRS). Models were adjusted for sociodemographic characteristics, health conditions, menopausal status, number of social roles. A final adjustment for depressive symptoms was made. Participants had Mage=47.14 (SD = 2.73) and most identified as non-Latina White (48.46%), spoke English (87.04%) and were perimenopausal (97.22%). On average, participants reported WHIIRS of 5.18 (SD = 3.89) and 31% had clinically significant depressive symptoms. Each additional role perceived as stressful was associated with a 0.09 unit increase in WHIIRS (SE = 0.01, p < 0.001). This association was attenuated when controlling for depressive symptoms (β = 0.05, SE = 0.01, p < 0.001). Our findings suggest that reducing stress from social roles may improve sleep quality among mid-life women. Future studies should leverage longitudinal designs to test the directionality of these associations.

